# A novel approach to monitoring lithium blood levels and renal function in patients receiving lithium during the COVID-19 pandemic

**DOI:** 10.1192/j.eurpsy.2022.964

**Published:** 2022-09-01

**Authors:** L. Gannon, J. Reynolds

**Affiliations:** 1 Lucena Clinic, Psychiatry, Dublin, Ireland; 2 Mayo University Hospital, Psychiatry, Castlebar, Ireland

**Keywords:** Lithium, Covid-19

## Abstract

**Introduction:**

Lithium is commonly administered to patients in an outpatient department (OPD) setting. Regular monitoring of lithium levels and renal function in accordance with published guidelines is required. In our unit, this is usually performed at OPD review. During the COVID-19 pandemic, reviews were either postponed or done remotely.

**Objectives:**

1. To devise a system to ensure that patients receiving lithium had appropriate blood test monitoring in the absence of traditional OPD appointments. 2. To assess the efficacy of this intervention by recording blood test dates and comparing with pre-COVID compliance.

**Methods:**

All outpatients receiving lithium, identified from the hospital database, received (1) a letter summarising the monitoring guidelines and (2) prospectively dated blood request forms. Patients at higher risk of contracting COVID-19 were advised to attend their primary care setting. Others were encouraged to attend primary care or our phlebotomy department. Compliance was measured by accessing the hospital’s laboratory enquiry computer based system and compared with pre-COVID-19 figures. Information was anonymised, as per General Data Protection Regulations.

**Results:**

57 patients receiving lithium were identified. Prior to the first Irish lockdown in March 2020, 16 (28%) were overdue testing. Three months into the pandemic, 15 patients (26%) were overdue testing.

**Conclusions:**

In the absence of routine outpatient appointments during the COVID-19 pandemic, the provision of written guidelines and completed blood request forms for patients receiving lithium was effective in ensuring monitoring of lithium levels and renal function. This system can be utilised as an alternative/adjunct to OPD review benefitting patients and health service delivery.

**Disclosure:**

No significant relationships.

